# Effect of Soil Type and Foliar Factors on the Distribution of *Imbrasia belina* in the Southeastern Lowveld of Zimbabwe

**DOI:** 10.1155/2018/9273184

**Published:** 2018-10-01

**Authors:** Edward Mufandaedza, Doreen Z. Moyo, Paul Makoni

**Affiliations:** ^1^Midlands State University, Department of Biological Sciences, P.O. Senga, Gweru, Zimbabwe; ^2^National University of Science and Technology, Department of Research and Innovation, P.O. Box AC 939, Ascot, Bulawayo, Zimbabwe

## Abstract

The aims of this study were to find out whether soil parameters (i.e., soil texture, soil pH, and available nitrogen (N) and phosphorus (P)) and level of tannins in the bark of tree as measured by total amount of N & P in the droppings significantly influenced *Imbrasia belina* distribution in the Southeastern Lowveld of Zimbabwe. The samples were collected in February-March 2013. Standard methods were employed on 80 samples across the four tenure regimes studied. Soil pH, percentage clay, silt, and sand were randomly measured across the four tenure regimes. The study results revealed that soil pH (*p*=0.475), % silt (*p*=0.172), % sand (*p*=0.907), available nitrogen (*p*=0.192), available phosphorus (*p*=0.247), and the mean tannin level (*p*=0.999) influenced the distribution of *Imbrasia belina* in the study area. Multiple comparison analysis showed that there were significant differences in percentage clay (*p*=0.044) between Gonakudzingwa Small-Scale Farms (GSSCF) and Chikombedzi Communal Area (CCA). However, Mwenezi Resettlement Area (MRA) and Gonarezhou National Park (GNP) results were insignificant for percentage silt (*p*=0.172) and percentage sand (*p*=0.907), respectively. The soil and foliar factors discussed are critical in determining *Imbrasia belina* distribution, forest health, and vitality.

## 1. Introduction

An important component of forest ecosystem is its ecological health status and the impact it has on sustainable growth. Evidence available suggests that new damaging agents are appearing at an increasing rate which could affect future sustainability of forest resources [1]. The effect of soil and foliar factors on distribution of *Imbrasia belina* is of critical importance so as to predict, map, and develop policy options for sustainable management as they follow their host *Colophospermum mopane* (mopane tree).

The mopane tree is a widespread and important woody species over much of southern Africa, between the Tropic of Capricorn and 10° south [2]. It is a xeric species of the savanna woodland forests, being found mostly on heavy-textured soils in flat valley bottoms such as Zambezi, Okavango, Limpopo, Cunene, Shire, and Luangwa [3, 4]. The mopane tree is indigenous to most of southern African states of Angola, Zimbabwe, northern Namibia, northern Botswana, Southern Zambia, Southern Malawi, Mozambique, and northern South Africa [4] and plays host to *Imbrasia belina* (*I. belina*) that plays an important role in the nutrition of rural communities as it is a good source of vital crude proteins (63.5%), crude fats (18%), carbohydrates 11.4 (g/100 g), minerals 3.5 (g/100 g), and 543 of energy (kcal/100 g) [5–7]. This helps to improve the nutritional capacities and ultimately reduces health deficiencies. This shows that *Imbrasia belina* had considerable potential for alleviating nutritional inadequacies in rural communities, thereby improving livelihoods of malnourished communities. A study by Timberlake [2] confirms that mopane tree distribution follows clay-rich soils, but it is not known what explains this relationship. Similar work by Shava [8] modelled the spatial distribution and diversity of dominant woody species in Zimbabwe. However, the study did not explain what informs mopane tree distribution. Therefore, this study sought to establish whether the mopane tree and hence *Imbrasia belina* distribution is influenced by soil and foliar parameters. The study hypothesized that *Imbrasia belina* distribution was significantly influenced by the soil and foliar factors, and specifically, whether soil texture (i.e., % sand, % silt, and % clay) and foliar analysis (i.e., available nitrogen (N) and phosphorus (P) in parts per million (ppm) in the soil), tannins (% catechin (CE)), soil pH, total amount of N and P in the droppings of *Imbrasia belina* and how these parameters influence *Imbrasia belina* distribution. The information is important in coming up with sustainable management decisions, which enhances planning and mapping of the natural resource.

## 2. Materials and Methods

### 2.1. Study Area

The study was conducted in the Southeastern Lowveld of Zimbabwe. The study area occupies the region 21°00′–22°15′S and 32°30′–32°15′E and covers about 300,000 hectares in extent [9]. The study was conducted in four different property regimes, namely, Gonarezhou National Park (GNP) (state property), Gonakudzingwa Small-Scale Farms (GSSCF) (private property), Chikombedzi Communal Area (CCA) (common property), and Mwenezi Resettlement Farms (MRA) (state and private property). MRA comprises Edenvale, Jabula, Nardice, and Ironwood farms [9]. The study site is exactly the same site the researchers conducted a study on the management of nontimber forest products harvesting with emphasis on the rules and regulations governing *Imbrasia belina* access in 2013 which was published in the Journal of Agricultural Research in 2015.

The study area falls in natural region 5 of Zimbabwe, and rainfall ranges between 400 and 500 mm per annum with average daily temperatures of 18° to 24°C [9, 10]. The study area experiences three climatic seasons: a hot dry period from August to October, a cold dry period from May to July, and a hot wet period from November to April. The altitude of the study area varies between 165 and 575 m above sea level [9, 11]. The soils are predominantly shallow sands of the siallitic group derived from sandstone [9, 12]. According to prior research by the same authors, mopane is the dominant tree species found in the study area in association with *Kirkia acuminata*, *Dalbergia melanoxylon*, *Adansonia digitata*, *Combretum apiculatum*, *Combretum imberbe*, *Acacia nigrescens*, and *Commiphora* species [9].

### 2.2. Soil and Plant Tissue Sampling

**Figure 1:**
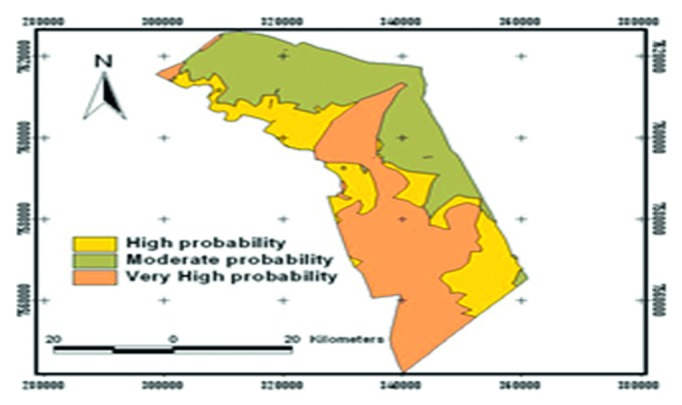
*Colophospermum mopane* tree probability map adapted from [8].

In this study, random sampling was used to establish sample locations across the four tenure regimes of Gonarezhou National Park, Gonakudzingwa Small-Scale Commercial Farms, Mwenezi Resettlement Area, and Chikombedzi Communal area based on [8] (Figure 1). The sampling procedures were done in very high, high, and moderate probability zones of finding *Imbrasia belina* as per the probability map given below. This enabled random sampling to be done in the woodlands giving each probability class an equal chance of being sampled.

A total of eighty (80) soil samples, *Imbrasia belina* droppings, leaf, and *Colophospermum mopane* bark samples were randomly collected from the four tenure regimes. Of these eighty soil samples, 20 samples of each parameter were randomly collected from a 10 m × 10 m plot in each tenure regime. Of the 20 trees randomly sampled in GSSCF, 15 were infested with *Imbrasia belina* whilst 5 had no sign of *Imbrasia belina*. Other tenure regimes had the following trees infected with *Imbrasia belina*: CCA had 12 trees infected and 8 uninfected; GNP had 8 infected and 12 uninfected; and MRA had 7 infected and 13 uninfected. An auger in combination with a spade was used to dig the soil to a depth of about 30–45 cm. The 20 soil samples from each tenure regime were collected and labelled in terms of tenure regimes, date, and the plot number on A4 khaki envelopes. The bark samples (20) from each tenure regime were collected on the *Colophosphermum mopane* tree at about 1.3 m above ground using a machete. The droppings of *Imbrasia belina* were collected under the *Colophosphermum mopane* tree found within the 10 m × 10 m plot. Bark and leaf samples were collected from the same tree, and these samples were further processed at the ICRISAT research laboratory, which is about 30 km from Bulawayo city, for analysis.

### 2.3. Soil pH Sampling Procedure

The pH of the soil suspension is a logarithmic device used to measure the acidity or alkalinity of the soil on a scale of 0 to 14, with 0 being acidic, 7 neutral, and 14 alkaline. The soil pH is expressed as the inverse log of the hydrogen ion concentration. The soil pH was determined according to the procedure described by Rhoades [13].

### 2.4. Determination of Soil Phosphorus (P)

The soil solution was extracted with 0.5 M solution of sodium bicarbonate at pH 8.5 [14]. The Olsen method is suitable for a wide range of soil types and pH values.

#### 2.4.1. Calculation of Phosphorus Concentration

The concentration of phosphorus in the sample was expressed in *P* mg/kg [14] as shown below:

(1) $$\begin{array}{c} P\left(\text{mg}/\text{kg}\right)=\frac{\left[\left(a-b\right)\times v\times f\times 1000\right]}{1000\times w}, \end{array}$$

where *a* = the concentration of *P* in the sample, *b* = the concentration of *P* in the blank, *v* = volume of the extracting solution, *f* = dilution factor, and *w* = weight of the sample.

### 2.5. Available Nitrogen (N) in the Soil-Colorimetric Determination of Nitrate

The determination of nitrate in the soil was done by extracting 0.5 M K_2_SO_4_ [15]. The following calculations were done to determine the solution concentrations for each unknown and the blanks. This is achieved by subtracting the mean blank value from the unknowns; this gives a value for corrected concentration (*C*) as shown below:

(2) $$\begin{array}{c} {\text{NO}}_{3^{-}}N\left(µ\text{g}/\text{g soil}\right)=\frac{\left(C\times V\right)}{W}, \end{array}$$

where *C* = corrected concentration (*µ*g/ml), *V* = extract volume (ml), and *W* = weight of the sample (g).

### 2.6. Soil Texture Particle Size Analysis

**Figure 2:**
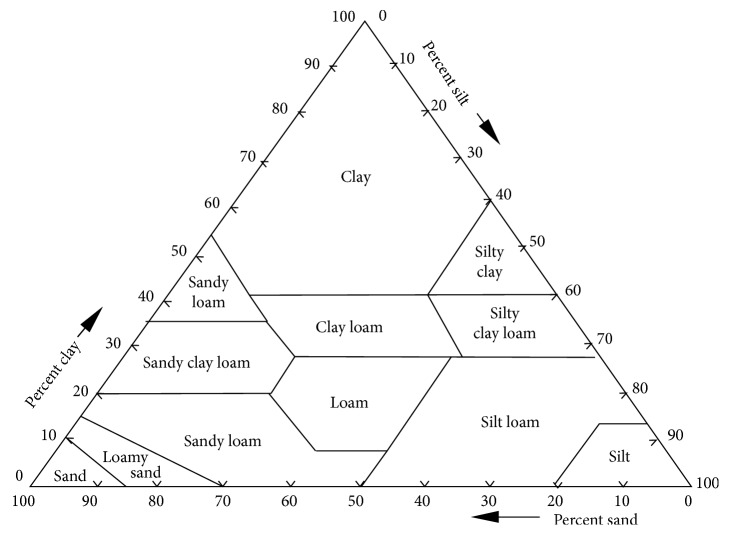
Soil textural triangle.

The soil texture particle size was analyzed by the Bouyoucos method [16]. The hydrometer was calibrated at (20°C), and correction factors were made in a temperature-controlled room which was kept at the correct temperature. Sand, clay, and silt percentage (%) calculations were performed by Bouyoucos [16]. After measuring soil texture distributions, the soil was assigned to a texture class based on the soil textural triangle (Figure 2).

Within the textural triangle are various soil textures which depend on the relative proportions of the soil particles. Users simply obtain the appropriate texture based on the particle size distribution.

### 2.7. Total Nitrogen and Phosphorus in (*Imbrasia belina*) Droppings

The percentage total N and P (droppings) represents the percent of total phosphorus and nitrogen in the droppings by weight. The content of total nitrogen and phosphorus was measured in a digest obtained by treating *Imbrasia belina* droppings with hydrogen peroxide + sulphuric acid + selenium + salicyclic acid [17].

### 2.8. Block Digester Procedure to Determine N and P in *Imbrasia belina* Droppings

The mean (0.3 ± 0.001 g) of oven-dried ground *Imbrasia belina* droppings (<0.25 mm, 60 mesh) at (70°C) were weighed into a labelled dry and clean digestion tube. 2.5 ml digestion mixture was added to each tube and the reagent blanks for each batch of samples using distillation-titration methods. The solution was made up to 50 ml with water, and the total N and P in the digests was determined as outlined below.

### 2.9. Determination of Total Nitrogen

Acid digestion of the *Imbrasia belina* droppings was achieved using the distillation-titration methods [17]. Free ammonia was liberated from the solution by steam distillation in the presence of excess alkali (NaOH). The distillate was collected in a receiver (50 ml conical flask) containing excess boric acid with drops of mixed indicator. An aliquot (5 ml for *Imbrasia belina* droppings) of the sample solution (digest mentioned above) was transferred to the reaction chamber of the still and added 10 ml of 1% NaOH. A steam distillation apparatus was set up using Markham or Hoskyn nitrogen still, and use of NH_3_-free distilled water wherever possible was achieved after the procedures of Anderson and Ingram [17].

Calculations of percentage *N* in *Imbrasia belina* droppings were performed:

(3) $$\begin{array}{c} \%N\text{ in }Imbrasia\;belina\text{ droppings}=\frac{\left[\left(a-b\right)0.2\times V\times 100\right]}{1000\times w\times \text{al}}, \end{array}$$

where *a* = volume of the titre HCl for the blank, *b* = volume of the titre HCl for the sample, *V* = final volume of the digestion, *w* = weight of the sample taken, and al = aliquot of the solution taken for analysis.

#### 2.9.1. Total Phosphorus without pH Adjustment Using Ascorbic Acid

5 ml of the supernatant clear wet-ashed digest solution was pipetted into a 50 ml volumetric flask. 20 ml distilled water was added to each flask. 10 ml of the ascorbic acid reducing agent was also added to each flask, beginning with standards (see below). The contents were made up to 50 ml with water, stoppered, and shaken well. The contents were allowed to settle for 1 hour to permit full colour development. The standards and sample absorbance (blue colour) were measured at 880 nm wavelength. Standards were pipetted according to the standard procedures.

Calculations to determine solution concentrations for each unknown and the 2 blanks were performed. The mean blank value was subtracted from the unknowns; this gives a value for the corrected concentration (=*c* in subsequent calculations):

(4) $$\begin{array}{c} P\text{ in sample}\left(\%\right)=\frac{\left[c\times v\times f\right]}{w}, \end{array}$$

where *c* = the corrected concentration of *P* in the sample, *v* = volume of the digest, *f* = dilution factor, and *w* = weight of the sample.

With a 10 ml digest aliquot (pH adjustment technique) and a 50 ml final dilution are used for colour intensity (absorbency) measurement,

(5) $$\begin{array}{c} P\text{ in sample}\left(\%\right)=\frac{\left[c\times 0.025\right]}{w}, \end{array}$$

where *c* = the corrected concentration for sample solution and *w* = the weight of sample taken.

#### 2.9.2. Vanillin-Hydrochloric Acid Method for Tannin Assays

The principle of the vanillin-hydrochloric acid procedure of tannin estimation is based on the Burns and Price methods [18, 19].

#### 2.9.3. Calculations of Tannin Assays

A standard curve was prepared by plotting the average absorbance readings of the duplicate determinations against catechin concentrations in mg/ml.

The sample blank absorbance was subtracted from the sample absorbance. The absorbance reading was converted into concentration of catechin/ml and calculated the catechin equivalents (CE) (%) as follows:

(6) $$\begin{array}{c} \frac{\left[\left(\text{mg catechin}/\text{ml}\right)\times \text{volume made up}\times 100\right]}{\text{volume of extract taken}\times \text{wt}.\text{ of sample}\left(\text{mg}\right)}. \end{array}$$

## 3. Results

There were no significant differences in soil pH across the four tenure regimes of Gonarezhou National Park (GNP), Chikombedzi Communal Area (CCA), Gonakudzingwa Small-Scale Commercial Farms (GSSCF), and Mwenezi Resettlement Area (MRA). The available nitrogen (N) and phosphorus (P) were found not to be significant. However, percentage clay was found to be significant whilst % sand, % silt, and tannin levels were found not to have an effect on the distribution of *Imbrasia belina*.  Table 1 shows the parameters measured, mean, ±standard error (SE), *F* value, and the significance level of the measured 80 samples in the four tenure regimes.

In this study, both the nitrogen (*p*=0.192) and phosphorus (*p*=0.247) were found not to be significant. However, the mean total percentage of nitrogen (*p*=0.659) and phosphorus (*p*=0.919) in the droppings of *Imbrasia belina* were also found not to be significant.

### 3.1. Soil pH

**Figure 3:**
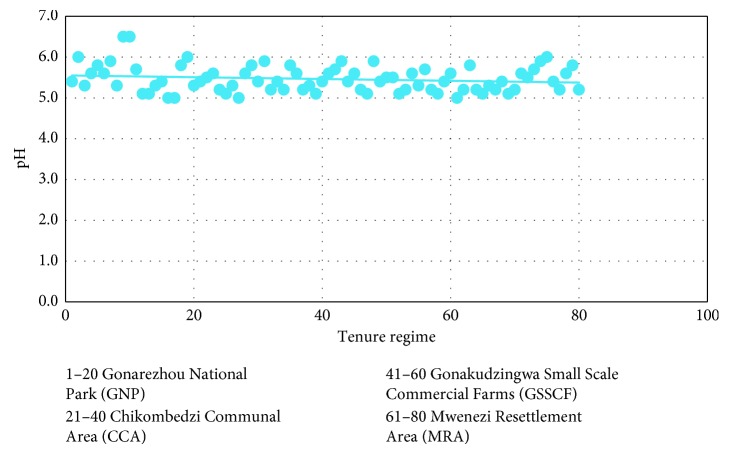
Tenure regime versus soil pH.

The soil samples results from the four tenure regimes of Gonarezhou National Park (GNP), Chikombedzi Communal Area (CCA), Gonakudzingwa Small-Scale Commercial Farms (GSSCF), and Mwenezi Resettlement Area (MRA) are presented in Figure 3. The soil pH (*p*=0.47) in the study area ranged between 5.0 M in Mwenezi Resettlement Area (MRA) and 6.5 M in Gonarezhou National Park (GNP).

### 3.2. Soil Texture

The mean, standard error, *F* value, and the significance level of the different soil textures from different tenure regimes are given in Table 1. Significant differences in the soil percentage clay (*p*=0.044) were observed between Gonakudzingwa Small-Scale Farms (GSSCF) and Chikombedzi Communal Area (CCA).

### 3.3. Available Nitrogen, Phosphorus, Total P Droppings to the Plants, and Condensed Tannin

**Figure 4:**
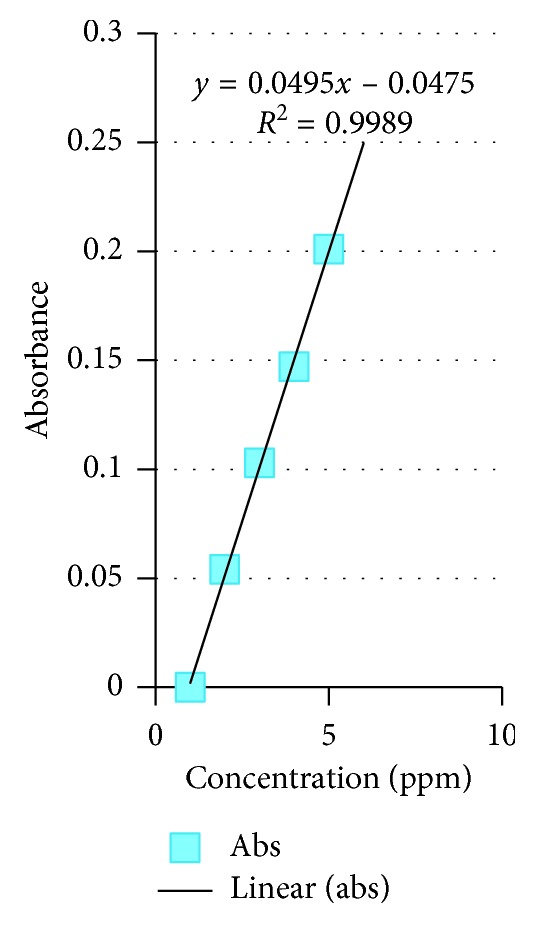
Available N standard graph.

**Figure 5:**
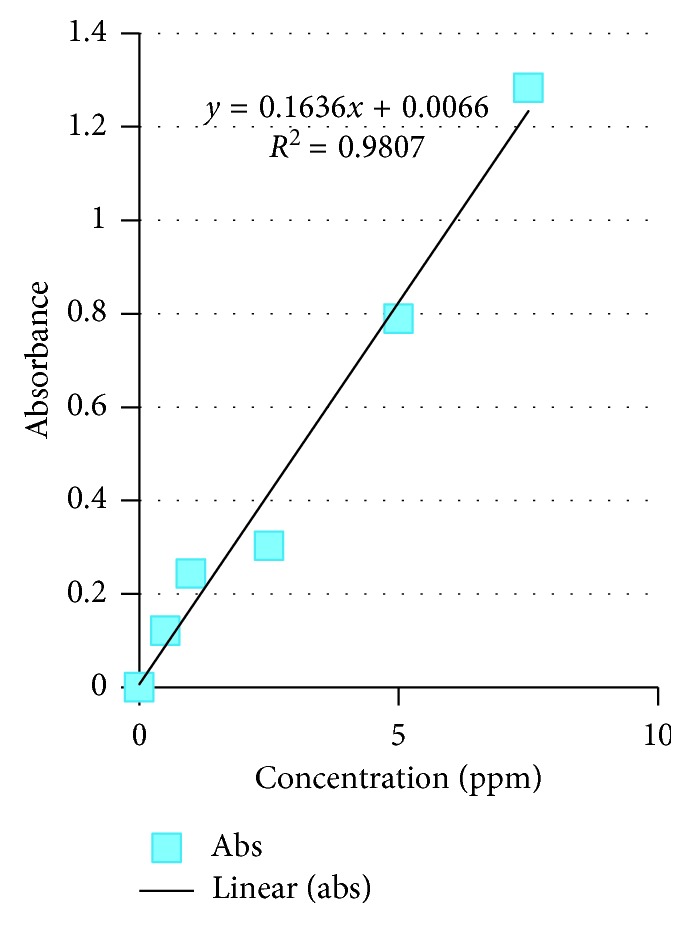
Available P standard graph.

**Figure 6:**
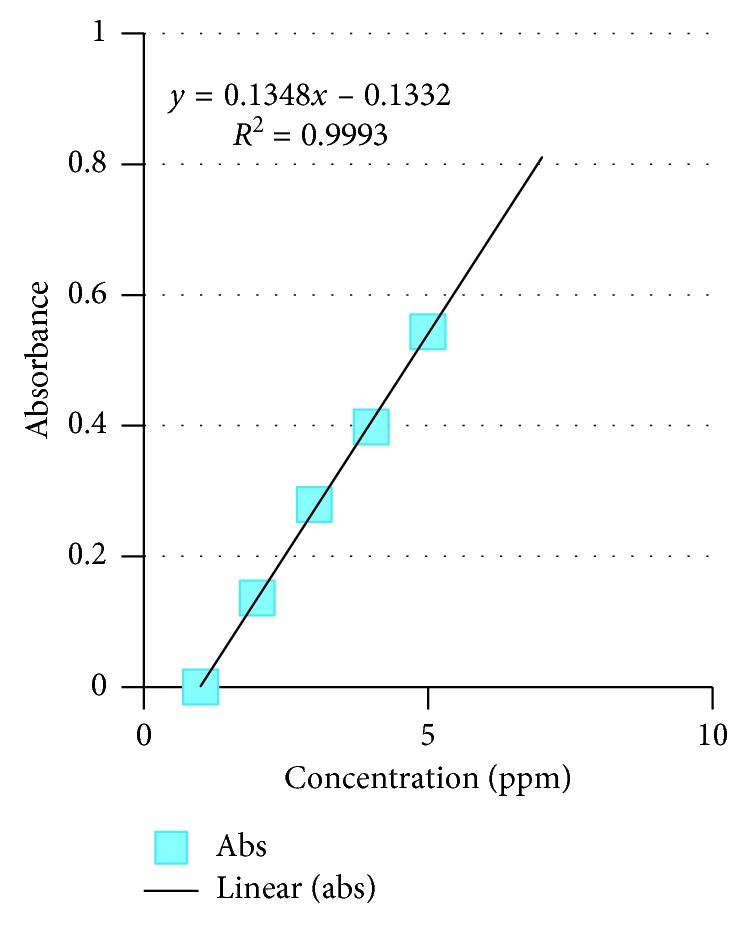
Total P droppings standard graph.

**Figure 7:**
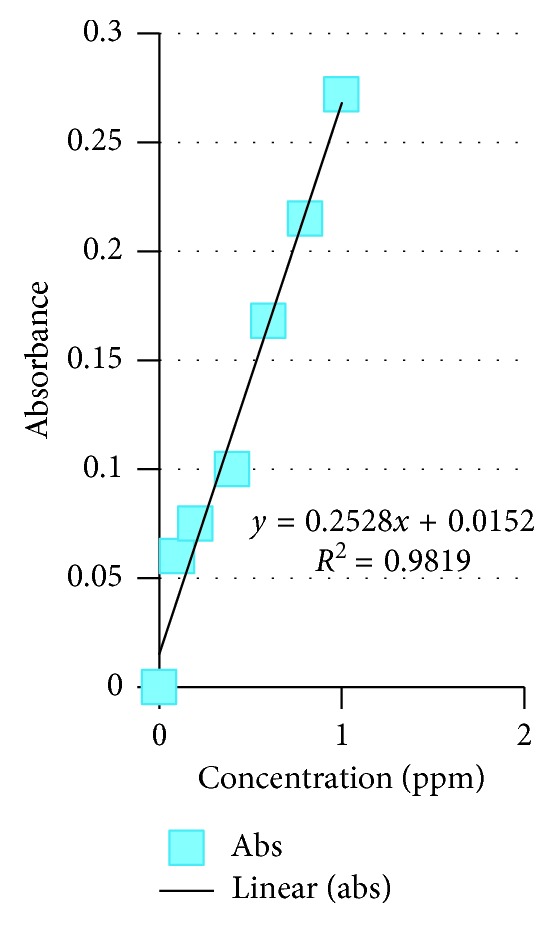
Condensed tannin standard graph.

The available nitrogen, phosphorus, total P, and condensed tannin of *Colophospermum mopane* in the four tenure regimes are shown in Figures 4–7. As for available N, the model output had *R*^2^ = 0.9989, for available P, *R*^2^ = 0.9807, total P droppings *R*^2^ = 0.9993, and for the condensed tannin *R*^2^ = 0.9819.

### 3.4. Standard Graphs for N, P, Total P, and Condensed Tannin

Standard graphs for N, P, total P, and condensed tannin are provided in Figures 4–7.

## 4. Discussion

The primary function of leaves in plants is to manufacture sugars and carbohydrates [20]. Sugars and carbohydrates are basic food or energy that plants use for all metabolic activities such as growth, root development, flower, seed production, disease resistance, and so on. Therefore, understanding the effect of soil and foliar analysis in *Colophospermum mopane* distribution and *Imbrasia belina* defoliation relations remains critical.

In this study, the percentage clay was found to significantly influence (*p*=0.044) the distribution of *Colophospermum mopane* and hence *Imbrasia belina* in Gonakudzingwa Small-Scale Commercial Farms and Chikombedzi Communal Area. This is in tandem with studies by Timberlake and Mapaure [2, 4] where *Colophospermum mopane* distribution was found to follow sodic soil conditions. This information is important to explain *Imbrasia belina* distribution in the study area. In a related study, Makhado et al. [21] found the distribution of *Imbrasia belina* to follow the low-lying areas of southern Africa's savannas. Studies by Hrabar et al. [22] had shown that more detailed tree characteristics, such as leaf size, shoot size, stem number, and even leaf chemistry (protein to tannin ratio and total polyphenols) had no influence on host choice. This is in line with findings of this research where tannin levels were found not to influence the distribution of *I. belina.*

However, the distribution of *Imbrasia belina* may not entirely be explained by the sodic soil conditions alone, but other factors should be looked at to give the total picture of what contributes to their distribution. One of the remarkable features of *Colophospermum mopane*, to note, reported by Timberlake [2] is its ability to form monospecific stands with an even-sized structure, a feature which, along with its readiness to coppice, lends it to *Imbrasia belina* distribution and aids woodland management. *Colophospermum mopane* generally occurs on clay-rich soils, but it is by no means confined to such soils alone [23–26] and indeed grows better on deeper soils [3, 4, 27].

*Colophospermum mopane* is a dry species of the savanna ecosystem of south tropical Africa, found mostly on heavier-textured soils in the wide, flat valley bottoms of lower altitude river valleys. It is an adaptable and successful tree species of south-central Africa, occurring over a wide range of ecological conditions, but being found principally in areas of lower agricultural potential and extensive land use. This behavior confirms with the results of this study and gives *Colophospermum mopane* an opportunity to expand its ecological range. There is a direct relationship between the management of *Imbrasia belina* and its distribution and how access is governed by the existence of institutional arrangements and common property management regimes [9]. A study by Timberlake [2] reported *Colophospermum mopane* occurring on sand overlying a clay-rich layer, such as found on the deep Kalahari sands and fossil drainage lines of western regions of Zimbabwe. Pockets of sodium-rich deep duplex soils particularly with 700 mm mean annual rainfall and above have a better chance of supporting the growth, perpetuation, and distribution of *Colophospermum mopane*.

The mean tannin levels across the four tenure regimes indicated that condensed tannins had no effect on the distribution of *Imbrasia belina*. The available nitrogen, phosphorus, total P, and condensed tannin of *Colophospermum mopane* in the four tenure regimes are shown in Figures 4–7. In a related study by Stack and others [28], it was observed that *C. mopane* does not contain hydrolysable tannin which is widely accepted as being the primary defense compounds against insects. This explains the close association between *I. belina* and *C. mopane*, while mopane woodland often recovers within a short period after defoliation with little mortality, and continuous defoliation may lead to three deaths [29]. A study by Silanikove et al. [30] reported that the major antinutritional effect of condensed tannin was reduction of protein availability and suppression of digestive tract enzyme activities. However, condensed tannin on its own cannot explain nutritive value of browse [31].

An explanation for the apparent lack of importance of foliar quality could be that *Imbrasia belina* have various evolved traits allowing them to handle the foliar chemical composition [32]. One such trait, for example, is that larvae may compensate for suboptimal foods by increasing their ingestion rate or duration of development [33]. Secondly, the larvae could have various physiological and morphological traits enabling them to exploit their host plant, such as the production of enzymes (in the gut or saliva) that reduce the detrimental effects of potentially damaging plant compounds [34]. A third trait applicable to *Imbrasia belina* caterpillars is their gregarious feeding behavior when young, as this is known to enhance the ability of herbivores to exploit their host plants [35].

In this study, it is suggested for further research that in-vitro digestibility and in-vitro gas production techniques could be explored to determine the nutritive value of *Colophospermum mopane* browse with respect to *Imbrasia belina*. The study found out that available P, N, total droppings, and condensed tannin did not significantly influence the distribution of *Imbrasia belina* in the study area.

## 5. Conclusions

The aims of this paper were to find out whether soil parameters (i.e., soil texture; soil pH, available nitrogen (N) and phosphorus (P)) and level of tannin in the bark of tree significantly influenced *Imbrasia belina* distribution in the Southeastern Lowveld of Zimbabwe. The results of this study showed that soil texture influenced the distribution of *Imbrasia belina* between Gonakudzingwa Small-Scale Farms and Chikombedzi Communal Area (CCA). This is in tandem with studies by Timberlake and Mapaure [2, 4] where *Colophospermum mopane* distribution was found to follow sodic soil conditions. However, the distribution of *Imbrasia belina* may not be limited to sodic soil conditions alone, but other factors may need further research to come up with a holistic list of factors that may contribute to their distribution.

## Figures and Tables

**Table 1:** Mean + SE, *F* value, and the significance level of the parameters measured.

Parameter	Number of samples (*N*)	Mean + SE	*F* value	Sig. level
pH	80	5.4312 ± 0.0369	0.843	0.475
Avail. N (ppm)	80	1.0086 ± 0.1598	1.621	0.192
Avail. P (ppm)	80	0.0004 ± 0.0001	1.407	0.247
% clay	80	84.7875 ± 0.4545	1.774	0.044^*∗∗*^
% silt	80	12.1375 ± 0.4414	1.709	0.172
% sand	80	2.9750 ± 0.1366	0.184	0.907
% CE	80	40.7434 ± 2.0373	0.01	0.9999
% total P	80	0.0933 ± 0.0030	0.166	0.919
% total N	80	0.2531 ± 0.0029	0.537	0.659

^*∗∗*^4.4% risk of concluding that a difference exists when there is no actual difference.

## Data Availability

The data supporting the findings of this study are available from the corresponding author upon request.
